# Erythroderma in the elderly

**DOI:** 10.1111/1346-8138.17538

**Published:** 2024-11-11

**Authors:** Toshiyuki Yamamoto

**Affiliations:** ^1^ Department of Dermatology Fukushima Medical University Fukushima Japan

**Keywords:** deck‐chair sign, inflammaging, papulo‐erythroderma, senile atopic dermatitis, Th2

## Abstract

Erythroderma is the end‐stage condition caused by various inflammatory diseases, presenting with widespread generalized coalesced erythema on the trunk and extremities. Erythroderma is not a disease itself, but rather is a symptom expressing erythrodermic condition, which is frequently associated with inguinal lymphadenopathy, chills, and mild fever. The clinical characteristics include sparing the folds of the trunk and extremities (deck‐chair sign), and cobblestone‐like disseminated grouping prurigo; however, the deck‐chair sign is not specific to papulo‐erythroderma (Ofuji disease). Erythroderma is induced by various causes, such as eczema, psoriasis, atopic dermatitis, drug eruption, lymphoma, lichen planus, pityriasis rubra pilaris, autoimmune bullous diseases, graft‐versus‐host disease, dermatomyositis, internal malignancy, and others. By contrast, it is not uncommon for even thorough investigations to often fail to identify any significant underlying or occult diseases. Such cases are often diagnosed as idiopathic erythroderma. In elderly cases, some regard erythroderma as late‐onset atopic dermatitis, even if the patient does not have a history of childhood atopic dermatitis, while others consider it as a distinct condition with immune responses similar to atopic dermatitis. The etiology of erythroderma is suggested to be a Th2‐dominant condition with IL‐4/IL‐13 playing a central role, suggesting that therapies targeting those Th2 molecules may result in sufficient effects. In this review, the characteristics of erythroderma in the elderly and new therapeutic approaches are discussed.

## 1 INTRODUCTION

Erythroderma (exfoliative dermatitis) is a condition presenting with coalesced diffuse erythema and pruritus.^1^, ^2^, ^3^ In the majority of cases, erythrodermic conditions cannot be improved with topical therapies and the course is typically chronic. Cases of erythroderma can be categorized into several groups: (i) initial conditions are easily detected, such as atopic dermatitis, psoriasis, or autoimmune bullous disease; (ii) causative diseases can be found after several examinations, such as drug eruption or lymphoma; and (iii) causative disorders are difficult to determine, even if detailed investigations are performed. Laboratory data often show increased levels of IgE and hypereosinophilia in the peripheral blood, which resemble signs of atopic dermatitis. Thus, it is easily speculated that the etiology of erythroderma favors a Th2‐type cytokine balance. However, whether erythroderma of the aged is a subtype of elderly‐onset atopic dermatitis is controversial. In this review, the characteristics of erythroderma in the elderly and new therapeutic approaches are discussed.

## 2 CLINICAL FEATURES

**FIGURE 1:**
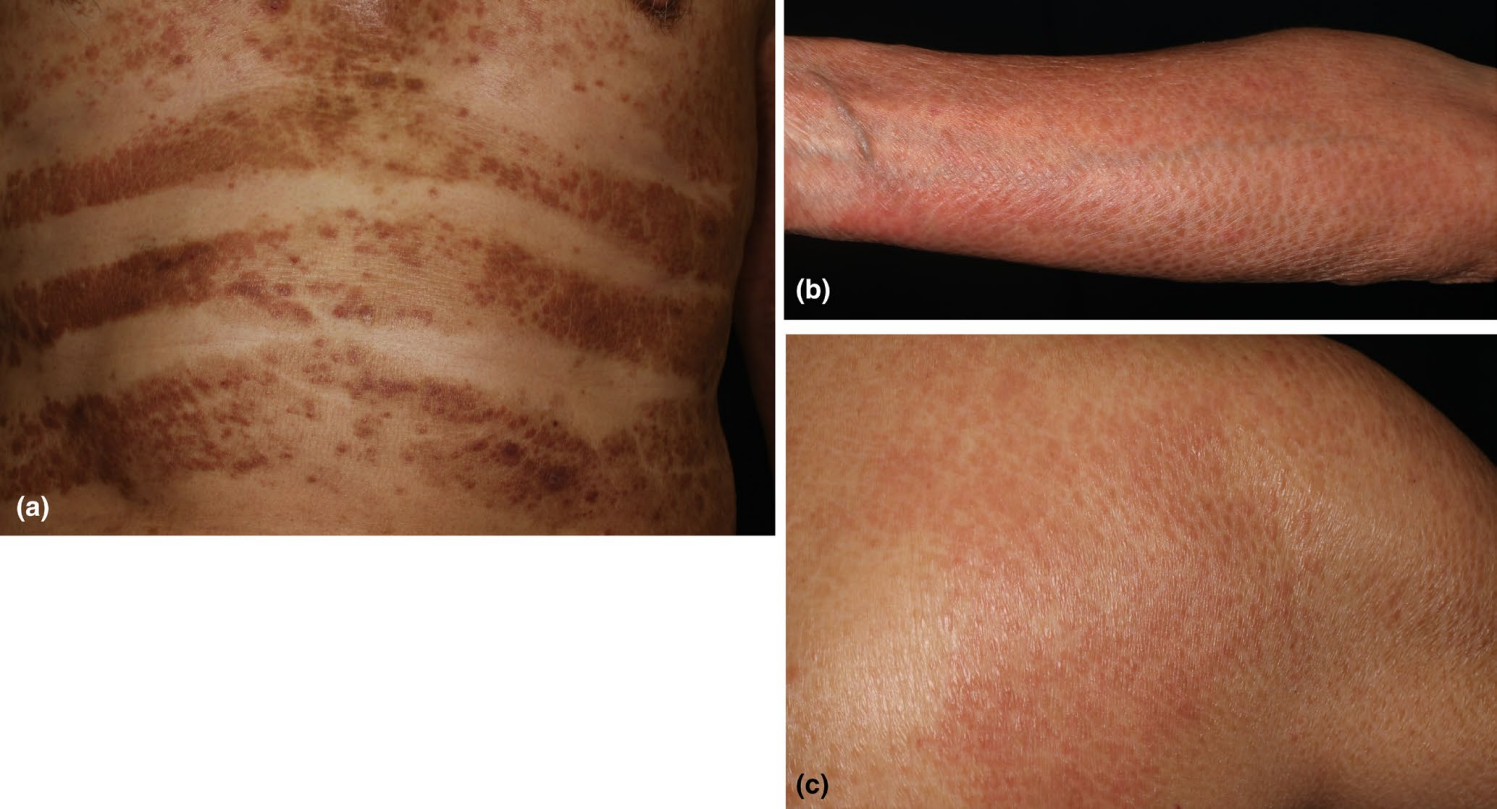
Deck‐chair sign sparing abdominal folds (a). Cobblestone‐like grouping prurigo on the upper extremity (b) and shoulder (c).

**FIGURE 2:**
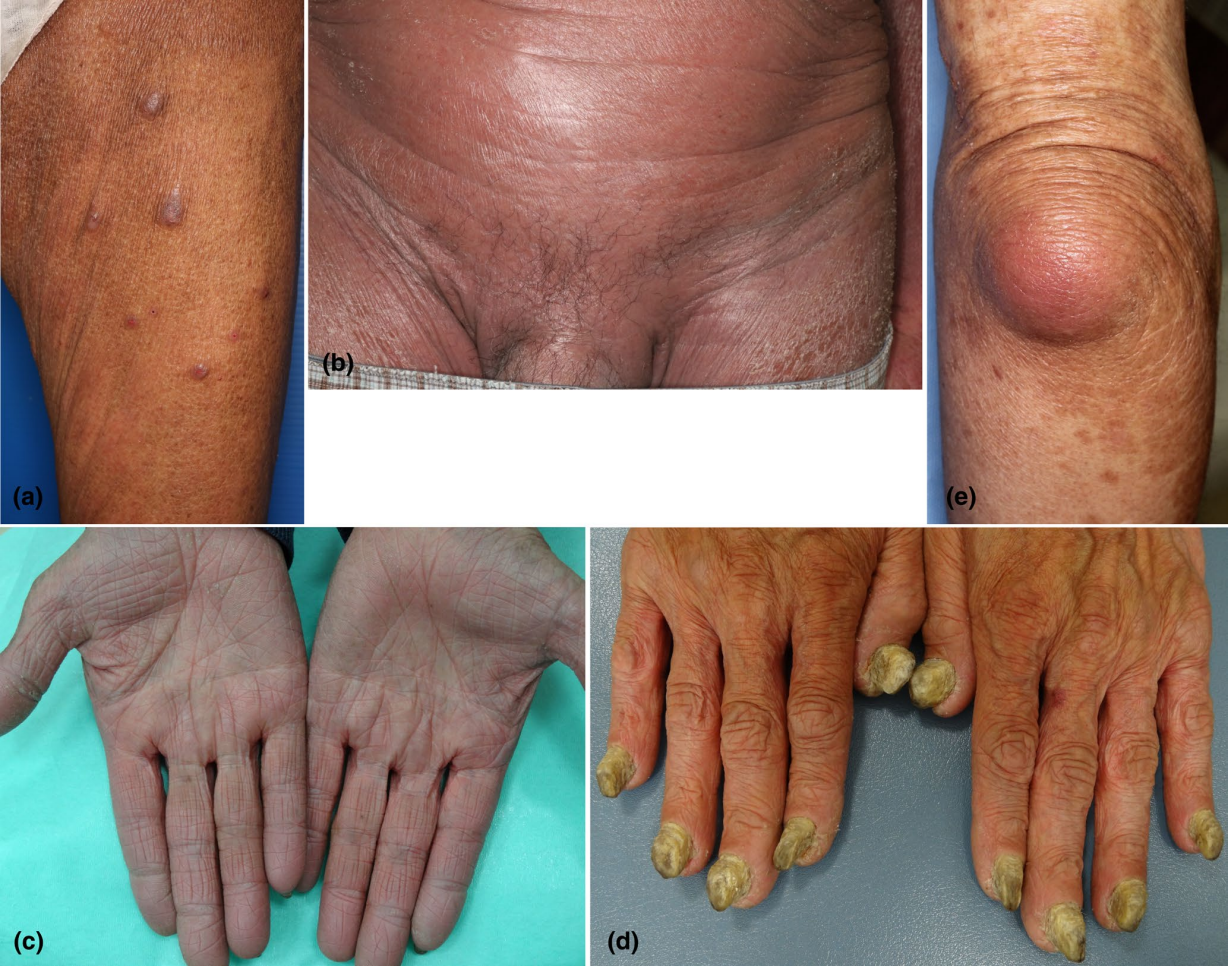
Erythroderma with pruriginous nodules (a). Loss of pubic hairs (b). Palmoplantar keratoderma (c). Thickening of all fingernails (d). Subcutaneous infection on the elbow (synovial bursitis) (e).

**FIGURE 3:**
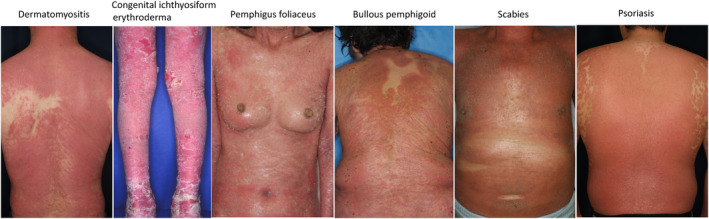
Various skin disorders developing secondary erythroderma.

Erythroderma presents with diffuse coalesced reddish or brownish erythemas involving >90% of the body surface areas of the trunk and extremities. The characteristic features are erythemas sparing the skin folds and creases of the trunk (i.e., abdomen or chest) and extremities (elbow fossa), as well as cobblestone‐like grouping prurigo (Figure 1a–c), but such features are not always apparent. Patients presenting with erythroderma frequently develop inguinal lymphadenopathy, chills, and mild fever. During the chronic course, the skin color becomes brownish, and nodular lesions, palmoplantar keratoderma, nail changes (thickening), hair loss of the scalp and pubis, and lower leg edema can be observed (Figure 2a–d). Furthermore, patients often develop dermal and subcutaneous infections (Figure 2e). The erythemas can be either well‐circumscribed or ill‐circumscribed, depending on the underlying conditions. Patients complain of severe itching; however, topical corticosteroids are usually ineffective. The diagnosis is easy based on clinical appearance, but after the diagnosis of erythroderma is made, exploration of the possible triggers, causes, and underlying disorders is important. When we encounter erythroderma patients, a number of investigations are required, including a detailed check of the patients' drug intake, lymphadenopathy, laboratory examination, skin biopsy, examination of internal malignancy, and others. Because erythroderma can be a non‐bullous form of bullous pemphigoid, biopsied skin samples should be cryopreserved, and sera also should be preserved. There are a number of cutaneous diseases which induce erythrodermic conditions, including eczema, psoriasis, atopic dermatitis, drug eruption, lymphoma, lichen planus, pityriasis rubra pilaris, autoimmune bullous diseases (pemphigus foliaceus, bullous pemphigoid), graft‐versus‐host disease, thymoma‐associated multiorgan autoimmunity, dermatomyositis, internal malignancy, chronic actinic dermatitis, Hailey‐Hailey disease, tinea corporis, scabies, COVID‐19 infection, and others (Figure 3).^4^, ^5^, ^6^, ^7^, ^8^, ^9^ By contrast, no underlying conditions related to erythroderma may not be detected even after thorough investigations. For such cases in which precipitating factors are undetermined, the term “idiopathic erythroderma” may be proposed. However, this should be regarded as a tentative diagnosis, and careful follow‐up is required to check for any potential underlying diseases that may emerge later because erythroderma is always secondary to other disorders.

## 3 PATHOGENESIS

Erythroderma is considered a Th2‐type cytokine dominant condition, and IL‐4 and IL‐13 are implicated to play important roles in the etiology of erythroderma. Erythroderma rarely presents with nodular lesions (prurigo nodularis), which are also Th2‐dominant condition. Biopsy specimens usually show normal or slightly acanthotic epidermis, as well as mild infiltration of mononuclear cells, CD4+ T cells, and eosinophils in the upper dermis. Depending on the stage, eczema, pruriginous papules (subacute prurigo), and pruriginous nodules (chronic prurigo) can be observed. In chronic courses, histiocytes are also seen in the biopsied skin. Infiltration of CD68+ and CD163+ M2 type macrophages has been observed in both idiopathic and secondary erythroderma, suggesting a potential role of M2 macrophages in both etiologies.^10^ Further studies are necessary to determine the possible roles of Th17 and innate lymphoid cell type 2 in the pathogenesis of erythroderma.

## 4 PAPULO‐ERYTHRODERMA (OFUJI DISEASE)

Papulo‐erythroderma (Ofuji disease) predominantly affects elderly males, and initially presents as lichenoid flattened papules, which sometimes form a grouping prurigo. In the original article, the characteristics are (1) predominant occurrence in elderly males, (2) eruptions consisting of firm, solitary reddish brown, dome‐shaped smooth‐surface nodules and diffuse reddish‐brown coalesced erythema of slightly elevated small patches, both of which have few scales, (3) sparing of the axilla, inguinal region, elbow and popliteal fossa, and abdominal folds, with the head and neck also unaffected, (4) absence of systemic symptoms other than pruritus, (5) histopathological features showing perivascular mononuclear cell infiltration containing eosinophils in the papillary to upper dermis, (6) presence of peripheral hypereosinophilia, and (7) a chronic course with gradual improvement.^11^ Although sparing of skin folds on the chest and abdomen, known as the deck‐chair sign, is frequently observed, it is not unique to papulo‐erythroderma. To diagnose papulo‐erythroderma, it is important to assess the clinical course, including the resistance of lichenoid papules and grouping prurigo to long‐term topical treatment, rather than solely depending on the presence of the deck‐chair sign. There are several speculations as to why skin folds and creases are spared, such as anatomical factors, sweat retention creating a moisture barrier in intertriginous regions, or prolonged retention of corticosteroids in skin folds; however, the exact mechanism remains unknown.

Although the etiology of papulo‐erythroderma is still obscure, an association with internal malignancy and drugs^12^ has been reported. IL‐4‐, IL‐13‐, and IL‐22‐producing T cells were markedly increased in the circulating peripheral blood of patients with papulo‐erythroderma.^13^ Papulo‐erythroderma was originally described in Japanese patients; however, there is a review paper from a foreign country from USA, in which atopy was present in 20.3% and malignancies were observed in 21.8% among over 100 cases.^14^ The authors proposed the following etiological classification of papulo‐erythroderma: (i) primary (idiopathic) papulo‐erythroderma, (ii) secondary papuloerythroderma for cases with a strong link to underlying causes, such as atopic diathesis, malignancies, infection, or drugs, (iii) papulo‐erythroderma‐like cutaneous T‐cell lymphoma (CTCL), in which the histopathology is consistent with CTCL, and (iv) pseudopapulo‐erythroderma for generalized rash without papules but positive deck‐chair signs.

## 5 PREVIOUS REPORTS OF ERYTHRODERMA

To date, there have been several reports on erythroderma collecting a number of cases in a single department or multicenter institutes (Table 1).^15^, ^16^, ^17^, ^18^, ^19^, ^20^, ^21^, ^22^, ^23^, ^24^, ^25^, ^26^, ^27^, ^28^ While the reported causes of erythroderma were diverse, it predominantly developed following pre‐existing dermatoses, with eczema, psoriasis, atopic dermatitis, drug eruption, and CTCL being the most common. The frequency of drug‐induced erythroderma varied between 1.5% and 46.6%. Erythroderma can be a paraneoplastic sign of malignancies, either internal solid cancers or hematologic malignancies, which have been observed in 0%–17.8% of patients with erythroderma. Hematologic malignancies include mycosis fungoides, Sézary syndrome, CTCL, adult T‐cell leukemia/lymphoma, and Hodgkin lymphoma.^26^, ^29^ The results of a study by our department showed that the incidence of malignancy associated with erythroderma was 10.8% (Irie and Yamamoto, unpublished). Idiopathic cases in which no significant underlying diseases were undetermined varied between 0% and 47.1%.

**TABLE 1:** Representative reports collecting over 50 cases since 2000.

	*N*	Idiopathic (%)	Preexisting dermatoses (%)	Drug (%)	Malignancy (%)
Sehgal, et al. (2004)^15^	80	22	58	20	0
Akhyani, et al. (2005)^16^	97	7.2	57.9	21.6	10.3
Rym, et al. (2005)^17^	80	7.5	72.5	11.3	8.8
Yuan, et al. (2010)^18^	82	6.1	68.3	17	4.9
Khaled, et al. (2010)^19^	82	25.6	43.9	21.9	4.87
Li, et al. (2012)^20^	260	14.2	70.8	12.7	2.3
Tan, et al. (2014)^21^	225	14.2	68.9	10.7	4
Nakano‐Tahara, et al. (2015)^10^	68	47.1	39.7	1.5	8.8
César, et al. (2016)^22^	103	3.9	65	18.4	11.7
Mathew, et al. (2017)^23^	370	15.7	74.6	6.5	3.2
Aqil, et al. (2019)^24^	92	0	43.5	38	17.4
Miyashiro, et al. (2020)^25^	309	16.8	Eczema (20.7), psoriasis (16.8), AD (8.7)	12.3	17.8
Long, et al. (2023)^26^	74	ND	85.1	4.1	5.4
Kondo, et al. (2024)^27^	88	2.3	48.9	46.6	2.3
Kliniec, et al. (2024)^28^	212	19.1%	Psoriasis (24.0), AD (13.2)	10.8	13.2

Abbreviation: AD, atopic dermatitis.

## 6 IS ERYTHRODERMA OF THE ELDERLY THE SAME AS ATOPIC DERMATITIS OF THE AGED?

Patients with erythroderma show the same serological pattern as those with atopic dermatitis, such as increased levels of IgE (RAST: radio allergo sorbent test and RIST:radio immunosorbent test) and thymus, and activation‐regulated chemokine (TARC) in the majority of cases. Ohga et al. reported that serum TARC levels in patients with chronic idiopathic erythroderma were significantly higher than those in patients with atopic dermatitis, whereas IgE levels were significantly lower, suggesting that the etiology of idiopathic chronic erythroderma may differ from that of atopic dermatitis.^30^ Tanei examined 60 patients with elderly atopic eczema and eczematous erythroderma was observed in 23.3%.^31^ Clinically, erythroderma differs from atopic dermatitis in several aspects, including male dominance and lack of lichenificated erythema on the cubital fossa.

Botella‐Estrada et al. analyzed 56 cases of erythroderma and concluded that patients with erythroderma of unknown cause can primarily be classified into three groups: those with senile atopic dermatitis, those with erythroderma related to internal or external medications, and those in a slow progression towards malignancies, mainly CTCL.^32^ Usually, atopic dermatitis develops in childhood; however, adult‐onset atopic dermatitis is not so rare, and phenotypic differences are observed between adult‐onset and child‐onset atopic dermatitis.^33^ Elderly‐onset atopic dermatitis or senile eczema is more significantly affected by environmental factors, such as air pollution, compared to early‐onset cases.^34^, ^35^, ^36^ Senile atopic dermatitis has been suggested to possess features of synergistic changes in the skin barrier and immune function.^37^ Cytokine profiling of the lesional skin of senile atopic dermatitis showed decreases in Th2/Th22 cytokines and increases in Th1/Th17 cytokines.^38^

## 7 INFLAMMAGING

Dysregulation of immune system functions associated with aging in humans is known as immunosenescence, which is influenced by factors such as innate immune response, genetic susceptibility, microbiota, and others. Immunosenescence is accompanied by an increase of proinflammatory symptoms and reduction of anti‐inflammatory milieu, and ultimately leads to low‐grade chronic inflammation, termed inflammaging.^39^, ^40^, ^41^ Inflammaging is associated with increased levels of proinflammatory cytokines such as TNF‐α, IL‐1α/β, IL‐6, IL‐8, and IFN‐γ. In addition, xerosis is induced by skin aging. The disruption of epidermal barriers increases the production of proinflammatory cytokines and inflammation in the skin. A skewed Th2 pathway during aging has been reported in mice^42^; however, it is unknown whether this occurs in humans. In addition, Th17/Treg imbalance^43^ and decrease in Th17 cells^44^ with age have also been reported.

## 8 NEW THERAPIES

It is difficult to control erythroderma, and uncontrolled severe erythroderma can be accompanied by electrolyte imbalance, dehydration, and hypoproteinemia, eventually leading to a life‐threatening condition.^45^, ^46^ Recently, several new drugs for refractory atopic dermatitis have been available. Therapies targeting Th2 type molecules, IL‐4 and IL‐13, may be expected for refractory erythroderma, although such therapies should be applied to idiopathic erythroderma after malignancies are excluded.

**FIGURE 4:**
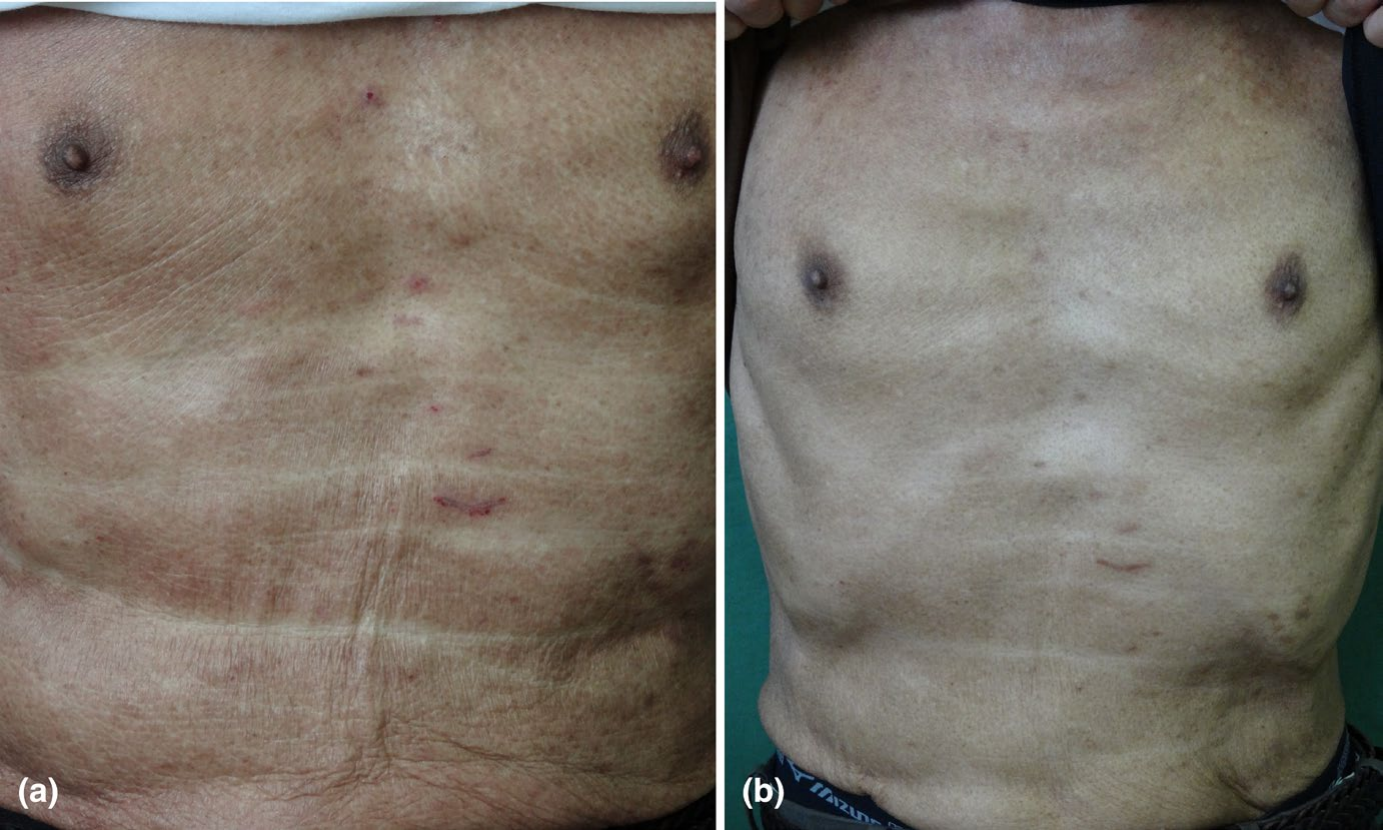
Erythroderma improved by dupilumab: (a) before administration and (b) after administration.

Idiopathic erythroderma is a chronic refractory diffuse erythematous condition of unknown causes and is resistant to various therapies. Dupilumab is a dual inhibitor of IL‐4 and IL‐13 receptors. Administration of dupilumab may lead to a Th1 cytokine balance by blocking Th2 cytokines. Several reports indicate that the administration of dupilumab has resulted in favorable and sufficient effects for papulo‐erythroderma^47^, ^48^, ^49^, ^50^, ^51^ (Figure 4a,b). Other new drugs, such as IL‐13, IL‐31, or JAK inhibitors, may also be expected. IL‐31 is a pruritogenic cytokine, belonging to the IL‐6 superfamily.^52^, ^53^ Nemolizumab is an IL‐31 inhibitor that targets IL‐31 receptor A and is effective for atopic dermatitis‐associated severe itching.^54^

## 9 CONCLUSION

There are several unresolved aspects regarding erythroderma. While idiopathic erythroderma predominantly affects elderly men, it should be clarified whether this is due to a shift in cytokine balance towards Th2 caused by aging or differences in cytokine balance based on gender. As a person ages, the risk of cutaneous xerosis increases due to decreased sweat gland activity, nutritional deficiencies (vitamin C, zinc), as well as changes in metabolism (energy expenditure).^55^ The onset of senile erythroderma may be attributed to internal and external environmental factors, such as low‐grade persistent inflammation, cytokine imbalance, impaired sweat function, decreased barrier function, individual predisposition, drug intake, topical corticosteroid use, and malignancy‐related factors. Further studies are necessary to elucidate the pathogenesis of this refractory condition.

## CONFLICT OF INTEREST STATEMENT

The author declares no conflict of interest.
